# An Analysis of 368 Cases of Pulmonary Tuberculosis

**Published:** 1913-01

**Authors:** 


An Analysis of 368 Cases of Pulmonary Tuberculosis examined at the Dispensary of the St. Paul Anti-Tuberculosis Committee, Leverett Dale Bristol, M. D., *St. Paul Med. Jour.*, Oct., 1912.

1. Of 368 positive cases only 67 (about 18 per cent) were in the earliest stage of the disease.
2. Only 27 per cent of the cases had a Family History of Tuberculosis.
3. Fifty-two cases had pleurisy in the past, 36 had pneumonia, and 19 influenza.
4. The average age was a little under thirty years.
5. The disease occurred slightly more in males (57 per cent) than in females (43 per cent).
6. The occupations of housewife, clerk, student, and maid, were those mostly involved.
7. The most common symptoms were cough and expectoration (76 per cent) and loss of weight (19 per cent).
8. The usual methods of treatment were employed.
9. About one-half of the patients had sanatorium or hospital treatment.
10. Glandular tuberculosis was far the most common complication.
11. 56 per cent of the patients died.
    8 per cent were greatly improved.
    7 per cent were apparently cured.
    5 per cent were unimproved.
    24 per cent left the city.

